# Isolated trisomy 7q21.2-31.31 resulting from a complex familial rearrangement involving chromosomes 7, 9 and 10

**DOI:** 10.1186/1755-8166-4-28

**Published:** 2011-12-05

**Authors:** Jörg Weimer, Simone Heidemann, Constantin S von Kaisenberg, Werner Grote, Norbert Arnold, Susanne Bens, Almuth Caliebe

**Affiliations:** 1 Department of Obstetrics and Gynaecology, Christian-Albrechts-University of Kiel, University Hospital of Schleswig-Holstein, Campus Kiel, Arnold-Heller-Str.3, Haus 24, Kiel, Germany; 2 Institute of Human Genetics, Christian-Albrechts-University of Kiel, University Hospital Schleswig-Holstein, Campus Kiel, Arnold-Heller-Str.3, Haus 10, Kiel, Germany; 3 Department of Obstetrics, Gynaecology and Reproductive Medicine, Hannover Medical School, Hannover, Germany

**Keywords:** chromosome microdissection, array-CGH, developmental delay, hypotonia, speech-delay, short stature, epicanthus, partial trisomy 7q21.2-7q31.31

## Abstract

**Background:**

Genotype-phenotype correlations for chromosomal imbalances are often limited by overlapping effects of partial trisomy and monosomy resulting from unbalanced translocations and by poor resolution of banding analysis for breakpoint designation. Here we report the clinical features of isolated partial trisomy 7q21.2 to 7q31.31 without overlapping phenotypic effects of partial monosomy in an 8 years old girl. The breakpoints of the unbalanced rearranged chromosome 7 could be defined precisely by array-CGH and a further imbalance could be excluded. The breakpoints of the balanced rearranged chromosomes 9 and 10 were identified by microdissection of fluorescence labelled derivative chromosomes 9 and 10.

**Results:**

The proband's mother showed a complex balanced translocation t(9;10)(p13;q23) with insertion of 7q21.2-31.31 at the translocation breakpoint at 9p13. The daughter inherited the rearranged chromosomes 9 and 10 but the normal chromosome 7 from her mother, resulting in partial trisomy 7q21.2 to 7q31.31. The phenotype of the patient consisted of marked developmental retardation, facial dysmorphism, short stature, strabism, and hyperextensible metacarpophalangeal joints.

**Discussion:**

For better understanding of genotype-phenotype correlation a new classification of 7q duplications which will be based on findings of molecular karyotyping is needed. Therefore, the description of well-defined patients is valuable. This case shows that FISH-microdissection is of great benefit for precise breakpoint designation in balanced rearrangements.

## Background

Phenotypic reports of chromosomal imbalances are an important source for genetic counselling especially in prenatal diagnosis. Chromosomal imbalances arise *de novo *or as the result of a familial rearrangement. The most common familial rearrangements are translocations. In case of an unbalanced segregation in an offspring the resulting imbalances consist of a combination of partial trisomy and partial monosomy. In most of the cases it is impossible to exactly relate the phenotypic consequences of an unbalanced translocation to either the underlying partial trisomy or the partial monosomy. Therefore many case reports are of limited value for genetic counselling because the phenotypic effects of trisomy and monosomy overlap [1]. Another difficulty in the description of phenotypic consequences of a certain chromosomal imbalance is the breakpoint designation. The precise description of the breakpoint is important for the genotype-phenotype correlation. In solely cytogenetically investigated patients, breakpoint designation remains doubtful due to the limited resolution of chromosome banding techniques. In recent years comparative genomic hybridisation (CGH) such as array-CGH has overcome many of the limitations of classical chromosomal banding analysis and can estimate the breakpoints with an accuracy of some kb. However, breakpoint designation by CGH and Array-CGH is restricted to unbalanced rearrangements. In case of balanced rearrangements or combinations of balanced and unbalanced rearrangements as in the present case further molecular cytogenetic techniques have to be combined with array CGH such as microdissection and Fluorescence-in-situ-hybridisation (FISH).

## Case report

**Figure 1:**
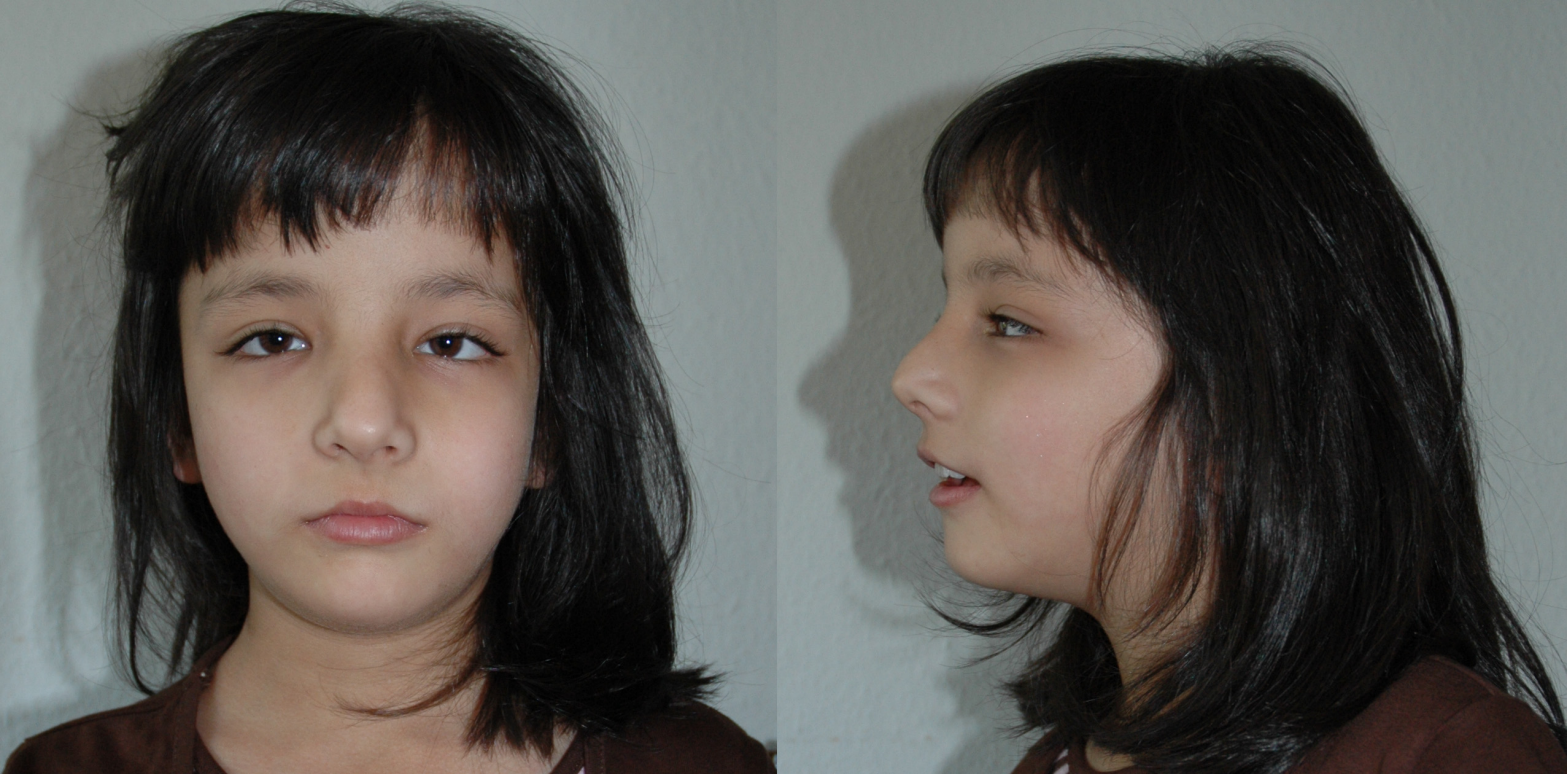
**The girl at the age of 7 8/12 years**. Note strabism, epicanthus, down-slanting palpebral fissures and slight hypertelorism.

The female patient is the first child of healthy non consanguineous parents. The father is German, the mother is from Pakistan. The family history was unremarkable. The girl was born spontaneously after an uneventful pregnancy at 39 weeks + 0 days with a length of 48 cm (- 1.3 SD), a weight of 2260 g (- 3.2 SD) and a head circumference of 34 cm (- 0.4 SD). The APGAR scores were 8/9/10 and the umbilical cord pH was 7.2. Due to muscular hypotonia nasogastral feeding had to be initiated. At the age of four months she was admitted to hospital due to repeated vomiting. At that time developmental delay was noted. At the age of five months frontal bossing, relative macrocephaly and strabismus were observed. With 5 3/12 years she started walking. During the last presentation at the age of 7 8/12 years she only spoke single words while, according to her parents, her receptive language skills were considerably better. There were no behaviour problems. At that time she was not continent yet. Her general health was good. Length was 112.5 cm (- 2.6 SD) and head circumference 51.5 cm (- 0.4 SD). The inner canthal distance was 3.3 cm (+ 2.0 SD). She had bilateral epicanthus and slightly down-slanting palpebral fissures. The previously noted strabismus had improved. The metacarpophalangeal joints of the fingers were hyperextensible. Due to recurrent ear infections she had received ventilation tubes twice. Generalised hypertrichosis was observed (Figure 1).

## Methods and Results

**Figure 2:**
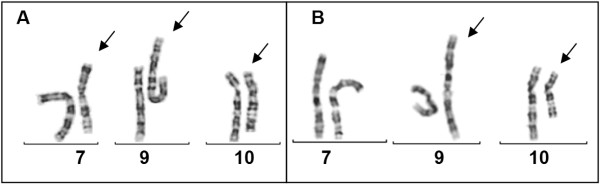
**Partial karyograms after GTG-banding**. **A **mother: 46,XX,t(9;10)(p13;q23)ins(9;7)(p13; q21.2-31.31) and **B **daughter: der(9)(10qter→ 10q23::7q21.2→ 7q31.31::9p13→ 9qter), der(10)(10pter→ 10q23::9p13→ 9pter) (according to ISCN 2009). The derivative chromosomes are marked by arrows.

Chromosome analysis in the girl was performed on peripheral blood lymophocytes according to standard techniques and revealed derivative chromosomes 9 and 10. Chromosome analysis of the parents revealed a normal male karyotype in the father and a balanced rearrangement t(9;10)(p13;q23)ins(9;7)(p13;q21.3q31.3) in 20 metaphases analysed (karyotype described according to ISCN 2009) in the mother. This unmasked the derivative chromosomes of the daughter as the result of a malsegregation of the complex maternal translocation (Figure 2): the girl inherited the derivative chromosomes 9 and 10 but a normal chromosome 7 from the mother resulting in isolated partial trisomy 7q21.3 to 7q31.3.

**Figure 3:**
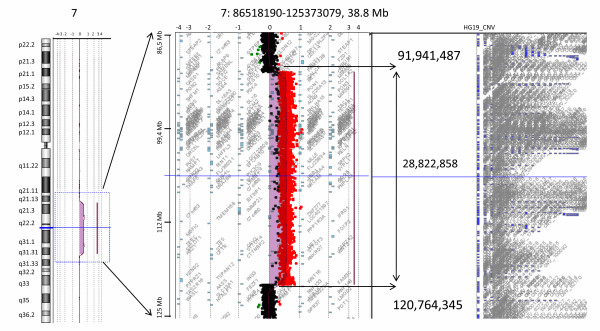
**Results of array CGH analysis using the Human Genome CGH Microarray 244A platform (Agilent Technologies, Santa Clara, USA), showing the internal boundaries of the duplication in 7q21q31.31 (91,941,487-120,764,345) and its exact size (28,822,858 Mb)**. The last normal oligonucleotide is 91,932,809 Mb and the first normal oligonucleotide is 120,770,258 Mb. The position of the array targets was mapped to the UCSC genome browser release February 2009 (GRCh37/hg19).

To estimate the chromosomal breakpoints of the derivative chromosome 7 and to exclude further imbalances we performed array-CGH from the patient's lymphocytes using the Human Genome CGH Microarray 244A platform (overall resolution 0,15 Mb, Agilent Technologies, Santa Clara, USA) according to the manufacturer's instructions. The array was scanned with the G2565CA Microarray Scanner System (Agilent Technologies, Santa Clara, USA) at a resolution of 5 μm/pixel. Signal intensities from the generated images were measured and evaluated with the Feature Extraction v10.7.3.1 and the Agilent Genomic Workbench Standard Edition 6.5 software packages (Agilent Technologies). By this analysis we detected a 28.82-28.83 Mb duplication of 7q21.2 to 7q31.31 (Figure 3) with the most telomeric duplicated probe starting at chr7:91,941,487 bp and the most centromeric duplicated probe ending at 120,764,345 bp (arr 7q21q31.31(91,932,809x2,91,941,487-120,764,345x3,120,770,258x2) (mapped according to GRCh37, hg 19) resulting in a revision of the breakpoint at 7q21.3 into 7q21.2 and in more detailed definition of the breakpoint at 7q31.3 into 7q31.31. Further chromosomal imbalances were not detected. A list of benign copy number polymorphisms can be obtained upon request.

**Figure 4:**
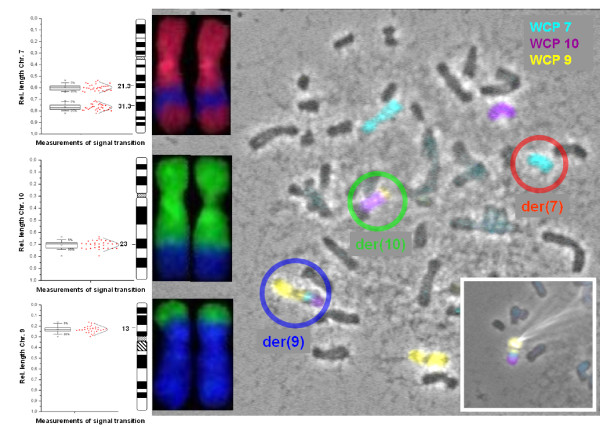
**FISH-microdissection of rearranged maternal chromosomes**. The origin of chromosomes was identified by whole chromosome painting probes (WCP): chromosome 7 (ice blue), chromosome 10 (purple) and chromosome 9 (yellow) are displayed and measured by the fluorescence and FISH Imaging System ISIS 3 (Metasystems, Altlußheim, Germany). The rearranged chromosomes of the balanced rearrangement are marked with circles. On the left side normal chromosomes are displayed hybridized with the labelled DNA from the microdissected chromosomes (reverse painting). Statistical analysis of the measured chromosome paintings was done using Microcal™ OriginR 6.0 (Microcal, Northampton,MA). On the very left side ideograms of the reverse painted derivative chromosomes are displayed to allow breakpoint designation.

To estimate the chromosomal breakpoints of the derivative chromosomes 9 and 10 these chromosomes as well as the derivative chromosome 7 were microdissected from chromosome preparations of the mother and rehybridised to normal human chromosomes [2]. In brief, to detect the chromosomal breakpoints spreads of the derivative metaphases of the mother were hybridised with three self made whole chromosome painting probes (wcp): the wcp probe for chromosome 7 was labelled with DEAC (Diethylaminocoumarin-5-dUTP; NEN Life Science Prod. Inc.; Boston, MA, U.S.A.), the wcp probe for chromosome 10 was labelled with R110-dUTP (Perkin Elmer; Waltham, MA, U.S.A.), and the wcp probe for chromosome 9 was labelled with Spectrum Orange-dUTP (Vysis Inc.; Downers Grove, Il, U.S.A.). Subsequently, the fluorescence labelled derivative chromosomes were isolated by a glass needle, amplified by DOP-PCR, labelled with three different fluorochromes and re-hybridised to normal human chromosomes. The microdissected chromosome 7 was labelled with R110-dUTP, the microdissected chromosome 10 was labelled with Spectrum Orange-dUTP and the microdissected chromosome 9 was labelled with Texas Red-12-dUTP. For better discrimination between the labelling of the wcp probes and the subsequent labelling of the microdissected chromosomes the rehybridised chromosomes were displayed in different colours (R110-dUTP in ice blue, Spectrum orange-dUTP in purple and Texas red-dUTP in yellow; Figure 4). The breakpoints of the derivative chromosomes could be identified by tracing back the labelled chromosomal segments to the ideograms of chromosomes 7, 9 and 10 (Figure 4).

Because of the complex maternal rearrangement amniocentesis was performed in a subsequent pregnancy revealing a balanced complex translocation in a male fetus. The boy was born at term with normal measurements (length 56 cm (+2 SD), weight 3020 g (-1.7 SD)). His motor development was normal. He started walking at the age of 11 months. At the age of 5 10/12 years length was 121 cm (0.74 SD). He attended preschool timely.

## Discussion

There are many publications on partial trisomies in 7q. In most cases the duplication resulted from a familial translocation involving the long arm of chromosome 7 and another chromosome leading to partial trisomy/monosomy 7 and partial trisomy/monosomy of the translocation partner, respectively [3-9]. About 19 patients with isolated trisomy 7 involving various regions of 7q have been described [10,11]. The phenotype varies according to the region which is duplicated and the size of the duplication. In an attempt to correlate the karyotype with the phenotype, patients with partial trisomy 7 have been divided into groups. Novales and co-workers suggested three groups [3]. Patients with a duplication 7q21 or q22 to 7q31 belong to group 1. The phenotype includes facial dysmorphism (frontal bossing, narrow palpebral fissures, epicanthus, and hypertelorism), strabism, hypotonia, and developmental delay. Group 2 includes patients with duplication 7q31 to 7qter. The phenotype is characterised by low birth weight, large fontanel, facial dysmorphism (narrow palpebral fissures, hypertelorism, small nose, low-set and malformed ears, microretrognathia), cleft palate, developmental delay, skeletal anomalies, and a reduced life expectancy. Group 3 is defined by a duplication of 7q32 to 7qter. These patients show low birth weight, facial dysmorphism (low-set ears, small nose, and hypotonia), kyphoscoliosis, skeletal anomalies, hypotonia and developmental delay. Courtens et al. described group 4 with a duplication involving 7q21 or q22 to 7qter [12]. One has to bear in mind that the clinical descriptions are mainly based on patients assessed by chromosome banding analyses.

The patient described herein has isolated partial trisomy 7q21.2 to 7q31.31 without additional chromosomal imbalances as confirmed by array-CGH. She therefore fits best into group 1 and displays the typical symptoms, namely low birth weight, global developmental delay with marked hypotonia in infancy, marked delay in speech development, mild short stature, normal head circumference, strabism and mild unspecific facial dysmorphism. Our patient can be best compared to the patients described by Humphreys et al. and Romain et al. [13,14]. Low birth weight was also a symptom in the patients described by Grace et al. and Berger et al. [15,16]. In contrast to other descriptions the palpebral fissures were of normal size.

## Conclusion

To enable a future new classification of duplications in 7q which will be based on findings of molecular karyotyping the description of well-defined patients is valuable. Furthermore, this case shows that FISH-microdissection is of great benefit for breakpoint designation in cases of balanced and/or complex rearrangements.

## Consent

Written informed consent was obtained from the parents of the patient for publication of this case report and all images.

## Competing interests

The authors declare that they have no competing interests.

## Authors' contributions

JW, SH and AC wrote the manuscript. JW carried out the FISH-MD and molecular cytogenetic analysis. CSvK did high resolution prenatal analysis of the patient. SB performed the array-CGH. AC performed cytogenetic analyses, clinical genetic investigation and genetic counselling. WG and NA coordinated the investigations.

All the authors have read and approved the manuscript.
